# Perception of Empathy in Mental Health Care Through Voice-Based Conversational Agent Prototypes: Experimental Study

**DOI:** 10.2196/69329

**Published:** 2025-05-07

**Authors:** Ruvini Sanjeewa, Ravi Iyer, Pragalathan Apputhurai, Nilmini Wickramasinghe, Denny Meyer

**Affiliations:** 1 Department of Health Science and Biostatistics, School of Health Sciences, Swinburne University of Technology, Hawthorn, 3122, Australia, 61 422587030; 2 School of Computing, Engineering & Mathematical Sciences, La Trobe University, Melbourne, Australia

**Keywords:** perceived empathy, mental health care, helpline service, conversational agent prototypes, voice interactions, demographic information

## Abstract

**Background:**

Empathy is a critical component of effective mental health care communication. Positive perceptions of empathy in conversational agents (CAs) operating in the health care domain are therefore needed to enhance the quality of care provided by these emerging technologies. However, research on how users perceive empathy in CAs is limited, particularly in voice-based prototypes.

**Objective:**

The objective of this study is to identify to what extent perceptions of empathy in CA prototypes correspond with the engineered empathy levels for these voice-based prototypes. In addition, as a secondary aim, this study investigates how the demographic characteristics of participants affect their perception of empathy in a mental health helpline service context.

**Methods:**

Swinburne University first-year psychology students (N=306) were presented with 9 CA prototypes engineered to portray low, medium, or high empathy levels, and their perceptions of empathy were collected via an electronic survey. Perceptions of empathy were rated using the Perceived Emotional Intelligence (PEI) Scale and the Raters’ Scale (RS10).

**Results:**

Most participants were female (233/306, 76%) with a mean age of 30 (SD 10.69) years, while a majority (194/306, 63%) were of Australian and New Zealand background. A strong positive correlation between the PEI and RS10 ratings was observed (*r*=0.829, *P*<.001). The empathy ratings across the 3 engineered empathy levels showed significant differences when using both PEI (*χ*^2^_2_=11.865, *P*=.003) and RS10 (*χ*^2^_2_=19.737, *P*<.001) measures. A linear mixed model for PEI showed significantly higher ratings for high rather than low engineered empathy levels (*t*_8_=−2.34, *P*=.048). RS10 ratings were also significantly higher for high rather than low engineered empathy levels (*t*_8_=−2.45, *P*=.04). However, no significant differences were detected between the CAs with engineered medium-level empathy and the CAs with low or high engineered empathy levels. The linear mixed model for PEI showed significantly higher ratings for participants of the Asian and Other ethnic categories compared to the Oceanic category (*t*_285_=2.54, *P*=.01 and *t*_286_=2.25, *P*=.03 respectively). The RS10 ratings were also significantly higher for the Other category rather than for the Oceanic category (*t*_284_=2.24, *P*=.03). Women showed significantly higher RS10 ratings than men (*t*_283_=1.94, *P*=.05).

**Conclusions:**

Recognizing empathy levels in CA prototypes proved challenging, highlighting possible complexities involved with voice-based empathy detection. The perception of empathy may also be affected by different ethnic and gender-based factors. The study findings emphasize the importance of personalized communications by CAs, with expressions of empathy tailored to key demographic characteristics of users. Future studies in a similar context would benefit from the inclusion of participants who are end users of a mental health care service with more balanced gender and age distributions. Multimodal interactions could also be considered for CA prototype development.

## Introduction

Empathy is a psychological construct that includes emotional as well as cognitive components. Empathy refers to the detection and perception of another person’s emotional experience, that is, feeling that emotional experience as one’s own. While empathy is critical for human interaction and the development of trust and safety [1], it is a complex and multidimensional construct [1]. Empathy requires an imagining of another’s experience, coupled with an interpretation of what they may be feeling and the reasons underlying these feelings [2].

Several factors moderate how individuals perceive empathy [3]. Women appear to perceive empathy more adeptly than men [4-6], while sensitivity to the emotional experiences of other people declines with age [6]. Cultural and ethnic backgrounds also influence how people perceive empathy [7]. Thus, social norms strongly inform how empathy is perceived and portrayed [8].

Conversational agents (CAs) with empathic capabilities offer advantages for mental health care [9], particularly in addressing current shortfalls in the current workforce [10]. If CAs can be shown to engage with users empathically, this technology may be leveraged to facilitate certain health care interactions, including patient triage, particularly via telephone and at scale.

In this experimental study, we engineer several vocal CA prototypes, displaying varying levels of empathy, which might feature in a triage context in a mental health helpline service setting. This study attempts to identify how perceptions of empathy in CA communications differ between individuals listening to staged conversations between a CA and a suicide helpline caller.

Although many studies have examined the concept of empathy [11,12], few studies have focused on how perceptions of empathy vary between individuals. An investigation of the perception of empathy by consumers of a customer service chatbot used the 3-item SERVQUAL scale to capture perceived levels of empathy. Based on feedback from participants recruited through electronic platforms, it was evident that empathy in chatbots leads to customer satisfaction at a level comparable with that achieved by human call-responders [13]. A second study proposed a new framework for evaluating empathy in dialogic systems using the Empathy Scale for Human-Computer Communication (EHSCC) in an attempt to standardize the concept of empathic assessment [14]. This framework is intended for a broader audience, not limited to clinical settings, where empathy assessments are made using text-based interactions or using transcriptions of voice-based interactions. A third study developed a Perceived Empathy of Technology Scale (PETS) to measure the perceived empathy of users with technologies such as chatbots [15].

This study differs from the aforementioned studies by using voice-based CA prototypes with varying levels of empathy, developed to reflect a mental health helpline service context. Swinburne University first-year psychology students, reflective of younger members of the broader community, were asked to evaluate the level of empathy perceived within each prototype using a range of assessment measures. Our study, therefore, extends the focus of previous research to the mental health care domain.

The primary objective of this study is to evaluate the extent to which ratings of empathy correspond with the levels of empathy engineered for each of the 9 CAs. The secondary objective was to determine how participant characteristics, specifically gender identity (sex assigned at birth), age, ethnic background, Aboriginal and Torres Strait Islander status, Index for Relative Socio-Economic Advantage and Disadvantage (IRSAD), and home language, may moderate perceptions of empathy.

Therefore, by investigating how engineered empathy in CA voice prototypes is perceived and whether the demographics of the participants impact this decision, the study offers insights into designing more effective empathic CAs for mental health care settings.

## Methods

### Data Collection

In this study, we engineered standardized vocal responses by 9 CA prototypes to short statements made by an artificial real-world caller to an Australian mental health telephone counseling helpline service. Short statements were identified by the research team from a large collection of call recordings obtained as part of a previous study [16]. Data collection was conducted through an electronic questionnaire developed in Qualtrics (see Multimedia Appendix 1), inviting participants to rate the level of empathy (eg, low, medium, or high) portrayed by each of 3 randomly assigned CAs.

### Participants

Participants were recruited from a Research Experience Program (REP) offered to first-year psychology students at Swinburne University of Technology, Australia. As a foundational unit of study provided at the undergraduate level within multiple disciplines (eg, science, humanities, and business), it is unlikely that the students would have any practical psychology experience, suggesting similar perceptions of empathy to the broader community. The advertisement for the study was posted on the internal REP platform housed on the university SONA system, which is accessible to this student group. Once interested students confirmed their decision to participate in the survey, they were provided with the survey link by the research administrator. Only students who were living in Australia and aged 18 years and older were eligible for participation.

### Survey Procedure

**Figure 1.**
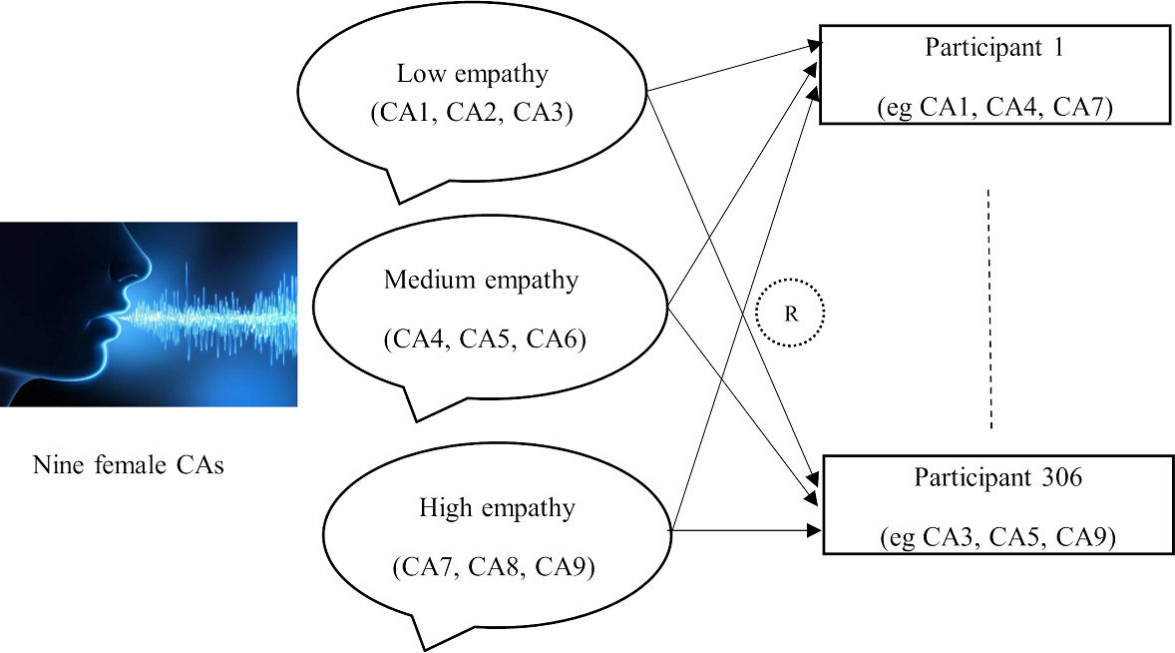
Outline of how the voice-based CA prototypes were assigned to participants in the study. CA: conversational agent; R: randomization. (reproduced with permission from Media Medic [https://www.mediamedic.studio] - All Rights Reserved.)

The survey was conducted between February 27, 2024, and June 2, 2024. Consenting students were electronically directed to the Qualtrics survey. Demographic information was collected from each student, including gender identity, age, residential postcode, Aboriginal and Torres Strait Islander status, ethnic background, and home language. Then, a set of 3 audio recordings was assigned to each participant as shown in Figure 1. These recordings were created as prototype CAs to be used within a mental health helpline service context. Each participant rated the level of empathy of each CA using two scales: the Perceived Emotional Intelligence (PEI) Scale [17] and the Raters’ Scale (RS10) [18].

This study has been reported in accordance with the Checklist for Reporting Results of Internet E-Surveys (CHERRIES) [19] shown in Checklist 1.

### CA Prototypes

The recordings for 9 prototype CAs were developed using the NaturalReader (NaturalSoft Ltd) [20], a speech synthesis software. Prototype responses were developed following an intensive analysis of counselor responses to callers of On The Line Australia (OTLA), a national telephone helpline counseling service. A range of only female voices was chosen, in part to simplify the experiment but also based upon focus group responses with OTLA staff, suggesting that female voices convey greater variation in empathy than male voices (blinded for review).

NaturalReader software allowed each voice to be modulated to accommodate either low, medium or high levels of conveyed empathy. The level of empathy chosen for each prototype was validated by research team members (blinded for review), drawing upon substantial experience working in mental health. The CA prototypes used in the study are mentioned under data availability.

### Measures

The level of empathy was collected using two measures: the Perceived Emotional Intelligence Scale (PEI) and the Rater’s Scale (RS10). The PEI is a 7-item scale with items rated on a 10-point sliding scale from 1=low empathy to 10=highest level of empathy. The RS10 is a single-item 10-point analog scale from 0=poor empathy to 10=highest level of empathy. This has proven reliable for researchers when minimizing the burden on participants to obtain responses within a short time.

A summary of the demographic features of participants and measures for perceived empathy used are attached in Multimedia Appendix 2.

### Statistical Analysis

RStudio (Posit, PBC, version 2024.04.2, Build 764) was used for all analyses. Participant characteristics and empathy ratings were summarized with appropriate descriptive statistics. Spearman rank correlation was used to assess the relationship between PEI and RS10 empathy ratings. Kruskal-Wallis tests were performed to evaluate the differences between the engineered empathy levels in CA prototypes in terms of participant ratings using the PEI and RS10 measures [21].

Empathy ratings were regarded as nested within individual participants. A random intercept model was fitted with the PEI rating as the response variable and the RS10 rating as a fixed effect to more accurately model the relationship between the PEI and RS10 ratings. A linear mixed model was fitted separately for PEI and RS10 ratings, with the participant demographic information and engineered empathy levels as the fixed effects. Random intercepts for each participant and each of the 9 CA prototypes were added. These models were designed to show how participant demographic information and engineered empathy levels impacted the empathy ratings provided by participants.

### Ethical Considerations

The study was approved by the Swinburne Human Research Ethics Committee (Ref: 20237339‐16599) and conducted in accordance with all aspects of the World Medical Association Declaration of Helsinki. The online survey clearly stated information about the study, its objective, what participation involved, and its benefits to participants, along with contact details of support services if a participant feels a low degree of discomfort due to the nature of the conversation included in the CA prototypes.

Consent was obtained as a part of the survey before proceeding to the questions. It was communicated to the interested students that participation was voluntary, allowing them to withdraw from the survey at any point. In addition, all responses were kept anonymous. The extracted survey results were stored in One Drive for Business with encryption accessible only to the research team. The students were not compensated for their participation. However, they received half a credit point for taking part in this 30-minute survey, which was a part of their course requirements. The credits were offered at the end of each week when the survey was closed for students who signed up that week to avoid any influence of the credit points on their engagement and responses. The study project was unrelated to first-year psychology course content, and survey completion was not a condition of receiving course credit; the REP program is a staple component of first-year psychology, in which most students choose to participate. Thus, we believe that any possible response bias was minimal and that the responses provided are not dissimilar to those that would be received from members of the general population within a similar age range. However, an attempt was made to validate the veracity of participant responses by checking the correlation between the PEI and RS10 ratings.

The details of the survey, including the consent form, are included in Multimedia Appendix 1.

## Results

### Participant Characteristics and Empathy Ratings

**Figure 2.**
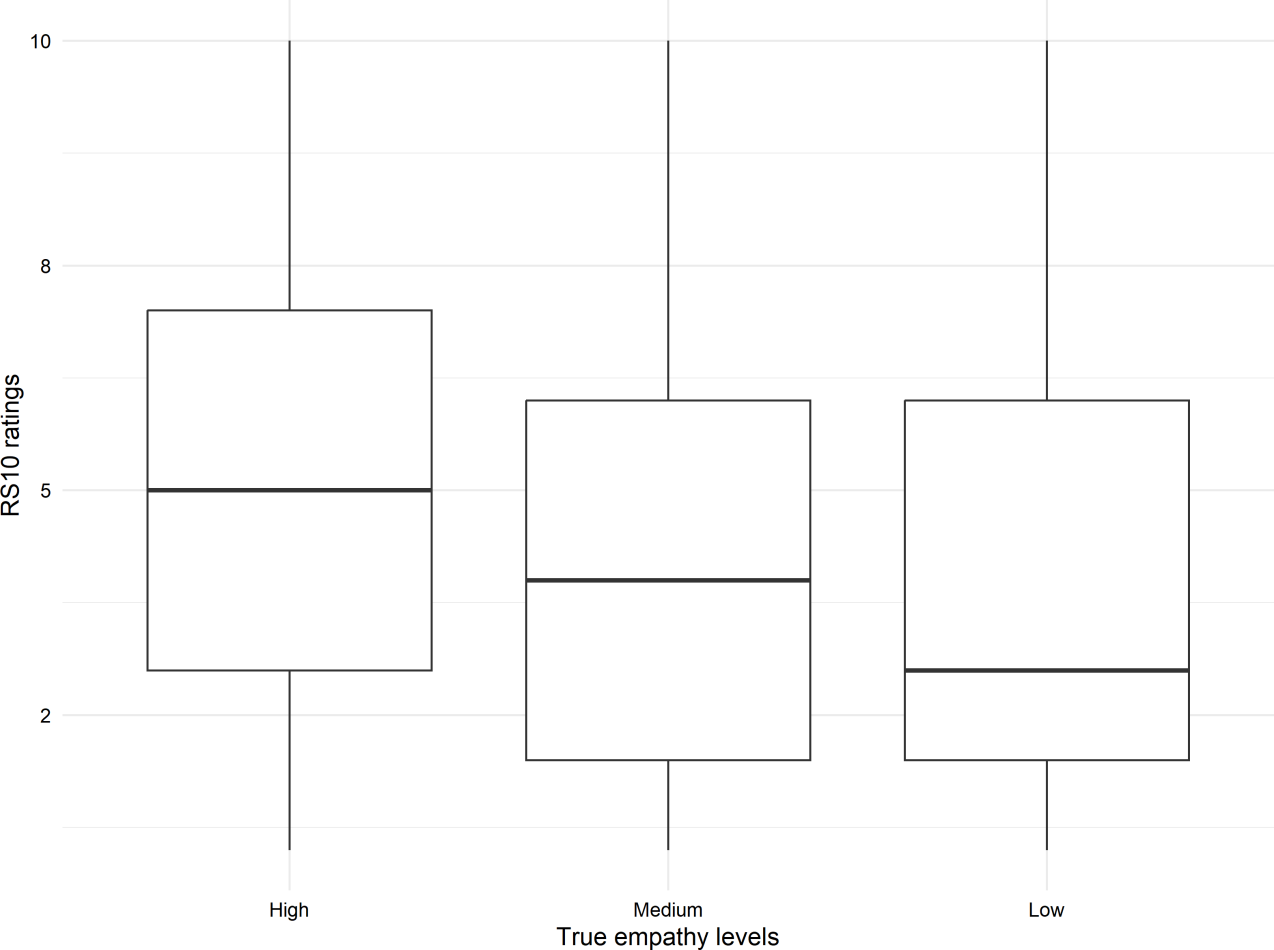
Distribution of RS10 empathy ratings by engineered empathy levels. RS10: Rater’s Scale.

Data were collected from 306 Swinburne University of Technology students. Participant characteristics are provided in Table 1. Ages ranged between 18 and 61 (mean 29.65, SD 10.69) years. A mean IRSAD decile (7.12, SD 2.62) was derived using participant postcodes [22]. Figures2  3 show the distributions for the RS10 and PEI empathy ratings according to engineered empathy levels.

**Table 1.** Summary of participant characteristics (N=306): demographic information.

Question	Participants, n (%)
Gender identity	
Woman	233 (76.1)
Man	58 (19)
Other	7 (2.29)
Missing	8 (2.61)
Do you identify as Aboriginal or Torres Strait Islander, or both?	
Yes	5 (1.63)
No	300 (98)
Missing	1 (0.33)
Is English your home language?	
Yes	258 (84.3)
No	47 (15.4)
Missing	1 (0.33)
Ethnic background	
Oceania (including Australia and New Zealand)	194 (63.4)
Asia	47 (15.4)
Europe	46 (15)
Other (America, Africa, and Middle East)	18 (5.88)
Missing	1 (0.33)

**Figure 3.**
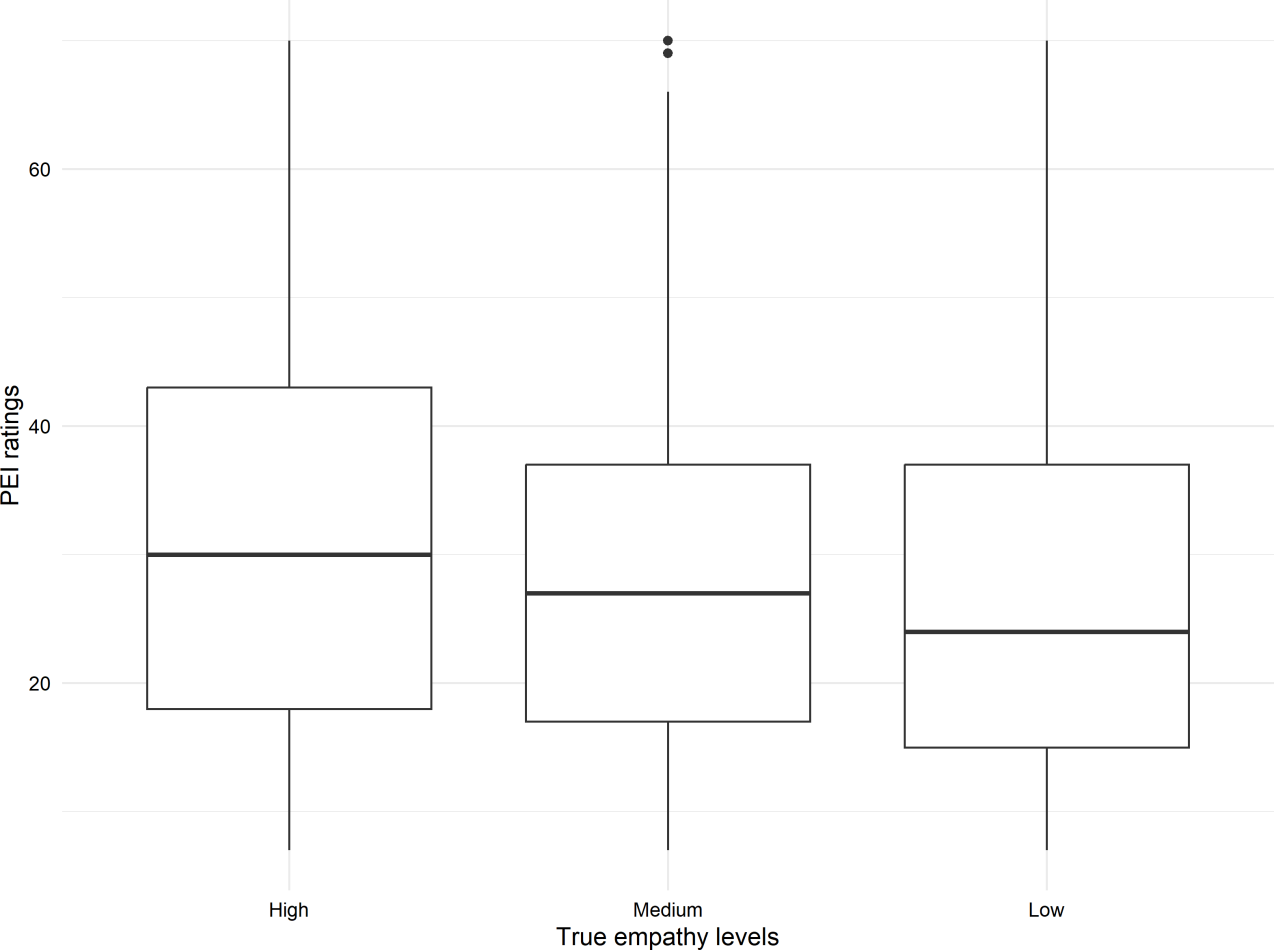
Distribution of Perceived Emotional Intelligence empathy ratings by engineered empathy levels. The two dots are outliers that are higher than the rest of the data points. PEI: Perceived Emotional Intelligence.

A strong positive correlation between the PEI and RS10 ratings was observed (*r*=0.829, *P*<.001), supporting the veracity of responses. Further validation was provided by a linear mixed model predicting PEI values from RS10 ratings (*β*=4.69, *t*_863_=45.8, *P*<.001). Ratings across engineered empathy levels revealed significant differences in both PEI (*χ*²_2_=11.86, *P*=.003) and RS10 (*χ*²_2_=19.74, *P*<.001) with higher ratings observed for the high empathy level compared to the low and medium levels.

### Linear Mixed Model Analyses

In the linear mixed model analyses, the percentages of variation attributed to participants were 63% and 46% for the PEI and RS10 ratings, respectively, while the percentages of variation attributed to the CA prototypes were only 1.5% and 2.5% for the PEI and RS10 ratings, respectively.

A linear mixed model was used to predict PEI ratings from engineered prototype empathy levels and participant characteristics (Table 2). PEI ratings were significantly higher for high rather than low prototype empathy levels (*β*=3.89, *t*_8_=−2.34, *P*=.048), as were PEI measures for participants of Asian rather than Oceanic ethnicity (*β*=6.53, *t*_285_=2.54, *P*=.01) and participants of the Other ethnicity category rather than Oceanic ethnicity (*β*=8.23, *t*_286_=2.25, *P*=.03).

**Table 2.** Linear mixed model for Perceived Emotional Intelligence ratings.

Fixed effects	Coefficient (β)	SE	*t* test (*df*)		*P* value
Intercept	26.68	4.67	5.72 (276.03)		<.001
Engineered empathy levels					
Low versus high	−3.89	1.66	−2.34 (7.79)		.048
Medium versus high	−2.65	1.66	−1.59 (7.79)		.15
Age	−0.06	0.07	−0.8 (286.71)		.43
Gender identity (woman vs man)	2.64	1.9	1.36 (285.34)		.17
Ethnicity					
Asia versus Oceania	6.53	2.57	2.54 (285.23)		.01
Europe versus Oceania	2.99	2.24	1.33 (285.72)		.18
Other^a^	8.23	3.66	2.25 (285.88)		.03
Is English your home language? (yes vs no)	0.58	2.63	0.22 (285.51)		.83
IRSAD^b^ decile	0.23	0.30	0.76 (287.01)		.45

a Sub-Saharan African, North African, the Middle East, and Peoples of Americas versus Oceania.

b Index for Relative Socio-Economic Advantage and Disadvantage.

A linear mixed model was also used to predict RS10 ratings from engineered prototype empathy levels and participant characteristics (Table 3). RS10 measures were significantly higher for high rather than low engineered empathy prototype levels (*β*=.82, *t*_8_=−2.45, *P*=.04), as were the RS10 measures for other ethnicities versus Oceania origin (*β*=1.18, *t*_284_=2.24, *P*=.03). In addition, women provided significantly higher RS10 values than men.

**Table 3.** Linear mixed model for Rater’s Scale ratings.

Fixed effects	Coefficient	SE	*t* test (*df*)		*P* value
Intercept	4.05	0.69	5.85 (218.18)		<.001
Engineered empathy levels					
Low versus high	−0.82	0.33	−2.45 (8.17)		.04
Medium versus high	−0.49	0.33	−1.46 (8.17)		.18
Age	0.01	0.01	−0.98 (284.94)		.33
Gender identity (woman vs man)	0.54	0.28	1.94 (283)		.05
Ethnicity					
Asia versus Oceania	0.65	0.37	1.75 (282.84)		.08
Europe versus Oceania	0.20	0.32	0.63 (283.6)		.53
Other^a^	1.18	0.53	2.24 (283.87)		.03
Is English your home language? Yes versus no	0.16	0.38	0.43 (283.34)		.67
IRSAD^b^ decile	0.03	0.04	0.76 (285.21)		.45

a Sub-Saharan African, North African, the Middle East, and Peoples of Americas versus Oceania.

b Index for Relative Socio-Economic Advantage and Disadvantage.

## Discussion

### Key Findings

This study aimed to investigate the complex issue of empathy perception and the factors that influence these perceptions. This novel study used experimental prototypes of voice-based CAs, engineered to reflect low, medium, or high levels of empathy in their vocals. The perception of empathy provided by study participants was compared against pre-established empathy levels. In addition, several demographic characteristics of the participants were evaluated to identify how these affected their perceptions of empathy.

The results indicate that perceptions of empathy do not always match with pre-established (“engineered”) empathy levels, and it appears that it is difficult for people to identify small differences in empathy levels (eg, between low and medium empathy and between medium and high empathy) using only vocal cues. In addition, it has been shown that demographic characteristics impact the way that people perceive empathy. In particular, ethnic background and to some extent gender have been shown to affect how people perceive empathy in this study.

The evaluation of empathy was conducted using two scales: the PEI scale, which is comprised of multiple factors catering to the broader concept of emotional intelligence, and the RS10 scale, a single-factor scale providing a more direct evaluation of empathy levels (0‐10). Most of the results from the linear mixed modeling were similar for the two scales, which provides more confidence in the conclusions. However, the participant gender effect was only marginally significant in the case of the RS10 rating and not significant in the case of the PEI rating, suggesting, perhaps, that the simpler RS10 scale is more sensitive to gender differences in perceptions of empathy.

Other studies have found that women have higher empathic ability compared to men [23], perhaps making them more attuned to the emotional status of others and, therefore, more likely to perceive empathy in others. Their higher empathic ability perhaps makes women more sensitive to empathic cues in others, leading them to provide more precise perception ratings. By far, most participants in this study were women, making it unfeasible for separate sex-based models to be developed. Future studies should try to obtain a more balanced representation of male and female participants so that an in-depth exploration of gender differences in empathy perception can be studied. In addition, a useful extension of this study would be to consider how nonbinary and other gender identities also perceive empathy, given that these minorities often suffer a greater mental health burden.

The PEI rating was perhaps more sensitive to the detection of ethnic differences than the RS10, because it detected significantly higher perceptions of empathy for participants with an Asian rather than Oceanic background, while the RS10 did not. As a measure of perceived emotional intelligence, the PEI measures a broader concept of empathy than the RS10. While empathy is the ability to put oneself in the place of another to understand their feelings [24], emotional intelligence is a set of skills that help us to effectively understand and express ourselves, understand and relate to others and cope with difficult situations [25]. The PEI scale’s multifactorial nature, therefore, evaluates a wider range of attributes than the RS10, perhaps explaining its greater sensitivity for detecting ethnic differences.

The ethnic background of the study participants had a significant impact on the perceived empathy ratings for both the PEI scale and the RS10 rating scale. Most participants were of Oceanic background (ie, Australia and New Zealand), which was also the reference group for the linear mixed model. When the PEI ratings were considered, participants from Asia and the Other category (Sub-Saharan Africa, North Africa, the Middle East and Peoples of Americas) provided significantly higher ratings than those of Oceanic ethnicity. However, only the Other category showed significantly higher ratings when using the RS10 scale. The demonstration of empathy is strongly informed by cultural background that reflects either individualist or collectivist values [26]. Western backgrounds are cited as more individualist, respecting and prioritizing individual rights and well-being more than non-Western backgrounds and less likely to see empathy in others. However, social desirability holds less importance in Western countries [27], perhaps resulting in more critical ratings (lower ratings) when it comes to perceptions of empathy compared to collectivistic countries [28], suggesting that differences in perceived empathy may be due to differences in social desirability.

These findings raise challenges for researchers trying to understand the complex phenomenon of empathy perception when designing an empathic CA. How does one design a CA that exhibits empathy for all end users when perceptions of empathy are so subjective, depending on gender and ethnic background? Also, what does this say about “true” or “engineered” empathy ratings? Maybe this helps to explain the lack of accuracy in the perceived empathy ratings for medium empathy levels that was discovered in this study and the absence of research addressing this crucial question.

### Limitations of the Study

The main limitation of the study was the poor generalizability of the sample of 306 participants who contributed to the electronic survey. The students from the REP were predominantly women (233/306, 76%). Most students in the sample were of Caucasian ethnicity from Australia or New Zealand (194/30,63%) and European background (46/306, 15%). In addition, only 5 of the 306 (1.6%) were of Aboriginal or Torres Strait Islander origin. Further, the students recruited for this study through the REP sample were mostly young adults, with 64% (197/306) within the age range of 18‐32 years, also having a relatively higher socioeconomic status with the average IRSAD decile. Therefore, future researchers are encouraged to extend the sample to individuals with a wider age range and level of socioeconomic status. Also, it is important that participants with more varied backgrounds are considered (not just psychology students), also allowing for more variation in ethnicity. It is also recommended that a suitable measure of social desirability will be included in future surveys, which will allow for adjustment of social desirability bias in the perceived ratings of empathy.

In addition, the sample consisted entirely of first-year psychology students. Future studies might benefit from a broader representation of the population that includes direct users of a mental health helpline service. It was not possible to access the direct users of a mental health helpline service within the scope of the current project. Instead, this project aimed to obtain a broad perspective on the perception of empathy in voice-based CA prototypes. The student participants were sourced from a range of fields including business, humanities, and science-based undergraduate courses and were therefore reflective of a broad range of life experiences and interests. Furthermore, the study population was aged between 18 and 32 years; among the most vulnerable group to experience mental health emergencies [29].

The perception of empathy is often based on more than just vocal cues. Nonverbal cues such as facial expressions and mannerisms can provide valuable supplementary information that conveys intention and context. It may be that particular ethnic groups and cultural backgrounds require more than just the CA vocalizations used in the present study.

### Future Opportunities

This study was focused on the design of a CA for deployment in a mental health environment, but these results hold broader implications because empathic communications are a key component of mental health care [30]. However, future research needs to acknowledge that the perception of empathy is a complex issue.

The significance of ethnicity as a predictor of perceived empathy suggests possible benefits in tailoring the training of health care professionals in the expression of empathy from a cross-cultural lens. Training guides for health care practitioners, teaching them how to adapt, demonstrate empathy, and personalize their communication styles based on gender and ethnic backgrounds, are also possibilities raised by this study. It is recommended that the development of these training materials be undertaken using a cocreation process involving relevant stakeholders.

The identification of CA empathy levels remains problematic. While our PEI and RS10 scales showed good agreement with each other and exhibited an ability to differentiate between low and high engineered empathy levels, they were unable to identify medium levels of empathy correctly. This might suggest a role for empathy education: promoting a greater understanding of how empathy is defined and reflected in speech. In addition, this study demonstrates that the display of empathy by CAs needs to be tailored to end-user characteristics. This will ensure that CAs used in various telephone mental health care applications can provide a more human-like user experience.

## Supplementary material

10.2196/69329 Multimedia Appendix 1 Online survey for evaluating conversational agent prototypes.

10.2196/69329 Multimedia Appendix 2 Summary of demographic features of participants and measures used for perceived empathy.

10.2196/69329 Checklist 1 Checklist for Reporting Results of Internet E-Surveys (CHERRIES).

## Abbreviations

CA: conversational agent
CHERRIES: Checklist for Reporting Results of Internet E-Surveys
EHSCC: Empathy Scale for Human-Computer Communication
IRSAD: Index for Relative Socio-Economic Advantage and Disadvantage
OTLA: On the Line Australia
PEI: Perceived Emotional Intelligence
PETS: Perceived Empathy of Technology Scale
REP: Research Experience Program
RS10: Raters’ Scale
