# Supplementary material for: Perception of Empathy in Mental Health Care Through Voice-Based Conversational Agent Prototypes: Experimental Study
Source: JMIR Form Res. 2025 May 7;9:e69329. doi: 10.2196/69329 (PMC12077854; doi:10.2196/69329)
Supplement: Multimedia Appendix 1 [file formative-v9-e69329-s001.docx]

Multimedia Appendix: Online Survey for evaluating CA prototypes

Empathy Survey

**Welcome to the empathy survey!**

**Title -**The evaluation of conversational agent prototypes.

 **Research Investigators:**

 Student Investigator: Ms Ruvini Sanjeewa,
 Email: rsanjeewa@swin.edu.au
 Telephone: 0422587030

 Chief Investigator: Professor Denny Meyer,
 Email: dmeyer@swin.edu.au

 Associate Investigator: Mr Ravi Iyer,
 Email: raviiyer@swin.edu.au

 **About the study**

This online empathy survey is part of a research study on developing a conversational agent (CA) able to exhibit empathy. The final CA will be called Alfi. Empathy has been described as 'the ability to understand and share in the feelings of someone else'. Past research has shown that the lack of empathy is one of the main factors preventing users from engaging with CA technology.
In this survey, we ask you to consider the performance of three CA prototypes that have been created to answer calls to a mental health helpline service. The role of these CAs is to gather essential information about the caller rather than to provide a counselling service. We invite you to provide your perceptions of the level of empathy portrayed by these three CAs.

 **Why is this study being undertaken?**
This survey is a part of the co-design process being used for the design of Alfi. This survey will tell us to what extent various Alfi prototypes portray empathy in their conversations with a helpline caller.
Your answers to the survey questions will help us understand how well the various Alfi prototypes are able to show empathy. We are only interested in the counsellor voices, so there are no questions that relate to the caller.

**Study and Researcher interests**
This study will form a part of the PhD study Ms Ruvini Sanjeewa is conducting.

Swinburne University of Technology is funding this PhD study.

**Who can take part?**
Only students enrolled in a first-year core psychology unit with Swinburne University of Technology or Swinburne online can participate. However, you should also be living in Australia, and you should be at least 18 years of age.

**What participation will involve?**
You will be asked several demographic questions designed to help us understand how the demographic characteristics of a listener affect perceptions of empathy. You will then be asked to listen to three fictional recorded conversations between three CAs ( Alfi prototypes) and a mental health helpline caller. You will be invited to rate the perceived empathy levels portrayed by each of these CAs and to provide

additional comments that assist us with the development of Alfi.
The survey will take 30 minutes, and you will receive a half-course credit point for participation.

**Risks & Benefits and Back-up support for participants**
Your participation will benefit your understanding of how psychology research is conducted while giving you the chance to be a part of such a project. Specifically, you will learn how to express empathy when dealing with a helpline caller.
Participants may experience a low degree of discomfort because the recordings include the voice of a caller discussing a recent hospitalisation to receive mental health support. If the recordings cause you any discomfort or distress, please contact Lifeline or Beyond Blue. **Lifeline Australia** – immediate mental health and crisis support, and suicide prevention – open 24/7 (phone); 7 pm-midnight AEST (online chat) www.lifeline.org.au  13 11 14.

 **Beyondblue** – mental health support – open 24/7 (phone); 3 pm-midnight, 7 days/week (online chat) www.beyondblue.org.au  1300 224 636.

**Participant consent and withdrawal processes**
It is up to you if you want to take part in this survey. You have the right to withdraw at any stage by emailing the researchers listed below. You can leave the survey by closing the browser. Should you leave the survey part way through, the researchers will retain the information you have already contributed, which may or may not form part of their analysis.

You will be providing written consent by signing the consent form before starting the survey. 

**Participant rights and interests - Privacy and Confidentiality**
All of your answers will be kept anonymous. All extracted data will be securely stored on One Drive for Business with an encrypted key and will only be accessible to research team members.
The data collected in this project will be retained indefinitely to answer the research questions of the original study or any related studies involving other researchers.
By starting this survey, you are giving extended consent for the data to be used for these purposes. Extended consent means that your data can be used in other research projects only after approval is given by the researchers listed below.

**Research Output**
The preliminary findings from the quantitative analysis conducted using the survey data will be shared with the survey participants upon request, in deidentified and summarised form. The findings of this investigation will be published in an academic journal.

 **Further information about the study - Who to contact?**
 If you would like further information about the study, please do not hesitate to contact:
**Chief Investigator: Professor Denny Meyer,
Swinburne University of Technology,
Burwood Road, HAWTHORN VIC 3122.
Email: dmeyer@swin.edu.au**

**Concerns/Complaints about the study - Who to contact?**
This project has been approved by or on behalf of Swinburne’s Human Research Ethics Committee (SUHREC), in line with the National Statement on Ethical Conduct in Human Research. If you have any concerns or complaints regarding the conduct of this study, you can contact:
**Dr. Astrid Nordmann,
Swinburne Research (H68),
Swinburne University of Technology,
PO Box 218, HAWTHORN VIC 3122.
Email: resethics@swin.edu.au
Telephone: 0392143845**

**The consent process:**

 I acknowledging that:
 a) My participation is voluntary and I am free to withdraw from the study at any time without explanation;
 b) I am at least 18 years of age, I live in Australia, and I am a first-year psychology student enrolled with Swinburne University of Technology or Swinburne-Online;
 c) I understand what I have read, including the potential risks of participation, and agree to take part in the processes described;
 d) I consent to the use of my personal information, as described.

 By signing this form, I agree to participate in this survey.

Q29 We hope you find this survey interesting!

Q1 To which gender identity do you most identify?

- Woman (5)
- Man (4)
- Other category (3)

| 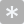 |
| --- |

Q2 What is your age in years? (e.g. 34)

________________________________________________________________

| 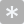 |
| --- |

Q3 What is your residential postcode? (e.g. 3024)

________________________________________________________________

Q4 Do you identify as Aboriginal and/or Torres Strait Islander?

- Yes (17)
- No (18)

Q5 Which ethnic background best describes you?

- Oceania (Incl. Australia and New Zealand) (23)
- North-west Europe (24)
- Southern and Eastern Europe (25)
- North African and Middle East (26)
- South-east Asia (27)
- North-east Asia (28)
- Southern and central Asia (29)
- Peoples of Americas (30)
- Sub-saharan Africa (31)

Q6 Is English your home language?

- Yes (1)
- No (2)

You will now be directed to three separate recordings of the CA, and each recording will be followed by the same set of questions relating to the performance of the CA.

Please listen to the attached recording and provide your answers to the following questions. Please note that Alfi is the name of the conversational agent you will be listening to.

1. Perceived Emotional Intelligence of Alfi
   Using this scale, we are trying to evaluate the extent to which Alfi demonstrates emotional intelligence. Emotional intelligence is the ability to perceive, use, understand and manage emotions. Remember, we are interested in the voice of the CA. These questions relate only to Alfi.

|  | 1=Never or  Almost  never true (1) | 2 (2) | 3 (3) | 4 (4) | 5 (5) | 6 (6) | 7 (7) | 8 (8) | 9 (9) | 10=Always or  Almost  always true (10) |
| --- | --- | --- | --- | --- | --- | --- | --- | --- | --- | --- |
| Does it seem that : Alfi listens openly to the caller’s emotions?   (1) |  |  |  |  |  |  |  |  |  |  |
| Does it seem that: Alfi can feel what the caller is feeling?   (6) |  |  |  |  |  |  |  |  |  |  |
| Does it seem that: Alfi understands the caller’s point of view?   (7) |  |  |  |  |  |  |  |  |  |  |
| Does it seem that: Alfi responds in a way that makes the caller feel that they are understood?   (10) |  |  |  |  |  |  |  |  |  |  |
| Does it seem that: Alfi is responding empathetically to the caller?   (11) |  |  |  |  |  |  |  |  |  |  |
| Does it seem that: Alfi is attempting to empathize with the caller?   (15) |  |  |  |  |  |  |  |  |  |  |
| Does it seem that: Alfi is thinking before responding? (16) |  |  |  |  |  |  |  |  |  |  |

1. How do you instinctively rate the overall level of empathy in Alfi's voice?

|  | 1=Low Empathy (1) | 2 (2) | 3 (3) | 4 (4) | 5 (5) | 6 (10) | 7 (11) | 8 (12) | 9 (13) | 10=High Empathy (14) |
| --- | --- | --- | --- | --- | --- | --- | --- | --- | --- | --- |
| The overall level of empathy observed in Alfi's voice from your perspective. (1) |  |  |  |  |  |  |  |  |  |  |

1. Please share further suggestions to improve the way Alfi interacted with the caller.

________________________________________________________________

________________________________________________________________

________________________________________________________________

________________________________________________________________

________________________________________________________________

1. What name would best suit the conversational agent?

- Alfi (4)
- Jamie (5)
- Other (7)

1. What would be your name suggestions for the conversational agent?

________________________________________________________________
