# Supplementary material for: Perception of Empathy in Mental Health Care Through Voice-Based Conversational Agent Prototypes: Experimental Study
Source: JMIR Form Res. 2025 May 7;9:e69329. doi: 10.2196/69329 (PMC12077854; doi:10.2196/69329)
Supplement: Multimedia Appendix 2 [file formative-v9-e69329-s002.docx]

Table 1: Summary of demographic features of participants and measures used for perceived empathy.

| Demographic features | Categories/ Range |
| --- | --- |
| Gender identity | - Woman - Man - Other category |
| Age in years | 18-32  33-46  47-61 |
| Index for Relative Socio-Economic Advantage and Disadvantage (IRSAD) | 1-10 (10=Highest advantage) |
| Do you identify as Aboriginal and/or Torres Strait Islander? | - Yes - No |
| Is English your home language? | - Yes - No |
| Ethnic background | - Oceania (Incl. Australia and New Zealand) - North-west Europe - Southern and Eastern Europe - North African and Middle East - South-east Asia - North-east Asia - Southern and Central Asia - Peoples of Americas - Sub-Saharan Africa |
| Perceived empathy measured using: | |
| 1. The Perceived Emotional Intelligence (PEI) scale [17] | A 7-item scale with each item rated from 1-10. Higher values indicate higher levels of empathy. |
| 1. The Rater’s Scale (RS10) [18] | A single-item Likert scale rating empathy from 0 to 10 with increasing levels of empathy. |
