# Supplementary material for: Perception of Empathy in Mental Health Care Through Voice-Based Conversational Agent Prototypes: Experimental Study
Source: JMIR Form Res. 2025 May 7;9:e69329. doi: 10.2196/69329 (PMC12077854; doi:10.2196/69329)
Supplement: Checklist 1 [file formative-v9-e69329-s003.docx]

Table 1: Checklist for Reporting Results of Internet E-Surveys (CHERRIES)

| Item Category | Checklist Item | Explanation |
| --- | --- | --- |
| Design | Describe survey design | The target population was a heterogeneous sample of first-year psychology students enrolled in various major degrees at Swinburne University of Technology. These students have some interest in psychology but little professional or life experience. They are, therefore, expected to provide a perspective on perceived empathy in voice-based conversational agent (CA) prototypes similar to what would be expected in a general population of first-year Australian students.  It is a convenience sample for which interested students voluntarily registered through the internal University research recruitment platform (SONA system). |
|  |  |  |
| IRB (Institutional Review Board) approval and informed consent process | IRB approval | The ethics approval for this study was obtained from the Swinburne Human Research Ethics Committee (SUHREC) before initiating the study. |
|  | Informed consent | The potential participants were informed about the details of the survey and what participation would mean on the cover page of the survey before electronic consent was obtained and before proceeding to the questionnaire. These details included the objective of the study, eligibility criteria, nature of the information requested from participants, potential benefits and risks to participants, how data would be protected, and contacts to reach out to in the research team and support services in case these were needed. |
|  | Data protection | Demographic details of the participants were collected from the participants. These data and the other participant responses were anonymous and were saved on One Drive for Business with encryption. Survey responses were only accessible to the research team. |
|  |  |  |
| Development and pre-testing | Development and testing | The online survey was developed by the main author in Qualtrics software, which was validated by the supervisor team before being finalised. The technical functionality, including random allocations of prototypes to participants, was cross-checked by a fellow researcher. These pilot tests were conducted before the survey was made available to the participants. |
|  |  |  |
| Recruitment process and description of the sample having access to the questionnaire | Open survey versus closed survey | This was a closed survey published on the University website open to any interested student who met the eligibility criteria (first year psychology students enrolled at Swinburne University of Technology in 2024 with an age of at least 18). |
|  | Contact mode | There was no initial contact with the participants since all interested participants joined through the SONA system. |
|  | Advertising the survey | The advertisement was prepared according to the University guidelines and posted online on the SONA system, accessible to all first-year psychology students. The survey advertisement is attached in Supplementary Materials. |
|  |  |  |
| Survey administration | Web/E-mail | The online survey was created using Qualtrics software, and the responses were automatically stored for extraction by the research team. |
|  | Context | The link to this survey was posted on the SONA system. Participants did not need to have Qualtrics software downloaded. The survey was made accessible to all users who were given access to the Qualtrics link. |
|  | Mandatory/voluntary | It was a voluntary survey. |
|  | Incentives | No monetary incentives were provided. However, half a credit point was awarded to each student who completed the survey for their course requirements. This half credit point was allocated to students who completed the survey by the SONA system at the end of each week. |
|  | Time/Date | During term 1 (From 27^th^ February 2024 to 2nd Jun 2024). |
|  | Randomization of items or questionnaires | Three of the nine prototypes were randomly allocated to each participant, ensuring that each student was presented with one low empathy, one medium empathy and one high empathy CA recording. The order of presentation for these three CAs was also randomised. |
|  | Adaptive questioning | The use of adaptive questioning was used under the CA name preference questioning. Participants who picked the other option had a question linked to provide their name suggestions. |
|  | Number of Items | There were 36 items across nine pages with no more than 8 items per page. |
|  | Number of Screens (Pages) | 9 pages. |
|  | Completeness check | The Qualtrics survey developed included a completeness check, with each item marked as a mandatory response except for the demographic information. Responses were not forced for the demographic questions. Additionally, the main researcher manually checked for the completion of survey outcomes before the analysis, excluding any surveys that were not completed. |
|  | Review step | The survey flow was designed to proceed forward without the option to go back for a review of answers. We needed to capture the immediate perceived empathy ratings, the first impression of each CA as experienced by the participants. |
|  |  |  |
| Response rates | Unique site visitor | In Qualtrics, a unique random ID was assigned to track each participant's response and avoid duplicates. This ensured the privacy and confidentiality of the responders. |
|  | View rate (Ratio of unique survey visitors/unique site visitors) | Unfortunately, we don’t have this information because the survey was anonymous. |
|  | Participation rate (Ratio of unique visitors who agreed to participate/unique first survey page visitors) | Not applicable. |
|  | Completion rate (Ratio of users who finished the survey/users who agreed to participate) | 320/324=98.7% |
|  |  |  |
| Preventing multiple entries from the same individual | Cookies used | The survey did not use cookies. Instead, duplication of unique response IDs was checked on the extracted data sheet, and there were none. |
|  | IP check | This survey did not implement tracking of the IP address of the user's computer to avoid duplicate entries from the same IP address. This was because it was possible that students used shared Swinburne computers for this purpose. The chances of repeating the survey for another 30 minutes were very low since only the initial completion would grant them half a credit point. Also, the thank you note at the end confirmed successful survey completion, so students knew when they had completed the survey successfully. |
|  | Log file analysis | Any repetition of demographic information among the responses was checked for uniqueness with regard to empathy ratings. |
|  | Registration | Students did register with the REP program before receiving the Qualtrics survey link. |
|  |  |  |
| Analysis | Handling of incomplete questionnaires | If a participant completed a prototype evaluation for only one or two CA prototypes out of the three that were randomised to them, these evaluations were retained in the final dataset. |
|  | Questionnaires submitted with an atypical timestamp | The University allowed the students a weeks’ time frame to complete their survey. Therefore, the expiration of the survey window was set to one week. However, the completeness of the responses was cross-checked before analysis. |
|  | Statistical correction | We removed categories that had a lower representation of the data, such as non-binary gender identity (2.29%) and Aboriginal and/or Torres Strait Islander origin (98% responded No). This decision was made to improve the reliability of the linear mixed model results. |
